# Indicators of Glucose Metabolism in Children and Adolescents Characterized as Having “Metabolically Healthy” and “Metabolically Unhealthy” Obesity

**DOI:** 10.3390/children12010050

**Published:** 2025-01-01

**Authors:** Maria Baltogianni, Niki Dermitzaki, Vasileios Giapros, Foteini Balomenou, Chrysoula Kosmeri, Fani Ladomenou, Evanthia Kantza, Anastasios Serbis

**Affiliations:** 1 Neonatal Intensive Care Unit, School of Medicine, University of Ioannina, 45500 Ioannina, Greece; mbalt@doctors.org.uk (M.B.); n.dermitzaki@uoi.gr (N.D.); f.balomenou@uoi.gr (F.B.); 2 Pediatric Department, School of Medicine, University of Ioannina, 45500 Ioannina, Greecefaniladomenou@uoi.gr (F.L.); kantzaevina@gmail.com (E.K.); aserbis@uoi.gr (A.S.)

**Keywords:** metabolically healthy obesity, children, adolescents, pediatric obesity, insulin resistance

## Abstract

Background/Objectives: Some individuals with obesity may exhibit fewer metabolic disturbances and face a lower long-term risk of complications; however, the existence of this so-called “metabolically healthy obesity” (MHO) compared to “metabolically unhealthy obesity” (MUO) remains controversial. We hypothesized that children with MHO might have a more favorable profile than children with MUO. Markers of glucose metabolism and insulin sensitivity were compared between children and adolescents diagnosed with MHO and MUO. Methods: This study recruited prospectively 104 children and adolescents (aged 6–16 years, 47 boys) with obesity. All participants underwent an oral glucose tolerance test (OGTT), and a comparative analysis was performed on HOMA-IR, QUICKI, insulin sensitivity index (ISI), insulinogenic index (IGI), disposition index (DI), and oral disposition index (oDI). Glucose metabolism indices were compared in these subgroups according to pubertal status. Results: Forty-seven children (45.2%) were diagnosed with MHO. The whole-body ISI differed significantly between the MHO and MUO groups (4.02 vs. 2.7, *p* < 0.01). The IGI was statistically lower in the MHO group compared to MUO (1.26 vs. 1.54, *p* < 0.01), while neither the DI nor the oDI differed significantly. A higher ISI (4.5 vs. 3.9, *p* < 0.01) was observed in prepubertal MHO individuals compared to MHO adolescents. Conclusions: Children classified as MHO according to the more recent criteria exhibit a more favorable metabolic profile than those with MUO. However, a completely healthy profile was not demonstrated in the MHO group, as many crucial metabolic profile parameters were comparable to those observed in the MUO group. The findings of this study indicate that all children with obesity, irrespective of whether they are categorized as having MUO or MHO, necessitate close monitoring.

## 1. Introduction

Childhood obesity represents one of the most prevalent and complex chronic diseases, with serious short- and long-term morbidities and an increased risk of early mortality [1]. Among the wide range of obesity-related morbidities, cardiovascular disease, type 2 diabetes, dyslipidemia, fatty liver disease, and depression are well-established consequences, with age increasing prevalence [1,2].

Obesity demonstrates significant heterogenicity, and it has been recognized that a subset of individuals with obesity does not exhibit the aforementioned cardiometabolic sequelae [3,4]. This phenotype is referred to as “metabolically healthy obesity (MHO)”, and in contrast to individuals with “metabolically unhealthy obesity (MUO)”, the metabolic profile is more favorable, characterized by normal blood pressure, lipids, and glucose metabolism.

MHO has been described since the 1980s and is generally defined in adults as the absence of components of the metabolic syndrome [5]. A universal definition of MHO in the pediatric population has yet to be established. It has been suggested that, since the metabolic syndrome is not clearly defined in children, childhood obesity should be classified as MHO or MUO based on the absence or the presence of a cluster of cardiometabolic risk factors, respectively [4,5,6]. A considerable variation is observed in the reported prevalence of MHO among youth with obesity [3]. In addition to heterogeneity in the criteria used to define MHO amongst studies, discrepancies in the definition of obesity and differences in study population characteristics are responsible for the wide variation in rates of MHO [3,4,7].

The MHO phenotype has been associated with sex, with an increased prevalence in females, possibly due to hormonal or lifestyle differences compared to males [3,7]. In addition, pubertal status has been shown to affect the occurrence of cardiometabolic disorders in obese children. Physiological changes, including increased insulin resistance during puberty, are potentially responsible for the decreased incidence of MHO in adolescents compared to younger children [4,8]. Few studies have examined the clinical and laboratory profile of children with MUO compared to those with MHO, while no study to date has compared glucose metabolism indices derived from the oral glucose tolerance test (OGTT) in these two groups of children and adolescents with obesity. We hypothesized that children with MHO should have less impaired indices of glucose metabolism compared to children with MUO.

The objective of this study was to compare markers of glucose metabolism and insulin sensitivity between children and adolescents diagnosed with MHO and MUO.

## 2. Materials and Methods

The registry of all children and adolescents aged 6–16 years who attended the Pediatric Obesity Outpatient Clinic of the University Hospital of Ioannina between January and December 2023 was reviewed to assess eligibility for inclusion in the study. All participants were instructed to avoid unhealthy dietary practices, such as consuming fast food or large amounts of pre-prepared meals, and to have their normal physical activity routine during the week prior to enrollment. The exclusion criteria for the study were as follows: children with chronic conditions not associated with obesity, such as genetic or endocrine disorders, autoimmune diseases, or other long-term illnesses, were excluded. Additionally, children with a recent acute illness, for example, infections of the respiratory or the gastrointestinal tract, or those taking medications such as corticosteroids, were also excluded. Lastly, children who had experienced significant (>10%) alterations in body weight within the previous three months, whether intentional or unintentional, were equally excluded.

The study protocol was reviewed and approved by the Institutional Scientific Review Board at Ioannina University Hospital. The parents of all participants were informed and provided consent.

### 2.1. Definition of MHO

Children and adolescents included in the current study were classified as having MHO or MUO in accordance with the criteria proposed by Abiri et al. in 2023. Based on the consensus, individuals characterized as having MHO should fulfill all of the following criteria: triglycerides (TGs) < 150 mg/dL, high-density lipoprotein cholesterol (HDL-C) > 40 mg/dL, systolic BP (SBP) and diastolic BP (DBP) < 90th percentile, and fasting glucose (FG) < 100 mg/dL. Conversely, children and adolescents were classified as having MUO if at least one criterion was not met [3].

### 2.2. Anthropometric and Clinical Assessment

An appointment was scheduled for all individuals eligible for inclusion in the study. A single pediatric endocrinologist conducted all anthropometric measurements and clinical evaluations (A.S.).

Height and body weight were measured, with the participants wearing light clothing and no shoes. A digital medical scale was used to measure weight to the closest 0.1 kg. A wall-mounted stadiometer was used to measure height to 0.1 cm accurately. Body mass index (BMI) was calculated as the ratio of body weight (kg) to the square of height (m^2^) and plotted on the WHO curves for BMI for age and sex. Values greater than +2 standard deviations (SD) defined obesity. Waist circumference was measured with a non-stretch tape measure at the midpoint between the last rib and the iliac crest, to the nearest 1 mm, with the participant in a standing position [9].

For each participant, blood pressure was measured three times from the left arm with a mercury sphygmomanometer after a 15-min period of rest in a sitting position, and the lowest value was recorded. Z-scores were calculated for both SBP and DBP [10]. Tanner staging was used to assess pubertal development [11,12].

### 2.3. Biochemical Assessment

All participants were required to fast overnight for at least 8 h before blood sampling.

Biochemical parameters were calculated for all participants using the Siemens Advia 1650 Clinical Chemistry System (Siemens Medical Solutions, Erlangen, Germany). HDL-C and TGs were measured. Fasting glucose and fasting insulin were measured, and the Homeostasis Model Assessment of Insulin Resistance (HOMA-IR) index and Quantitative Insulin Sensitivity Check Index (QUICKI) were calculated. Specifically, the HOMA-IR index was calculated as the product of the FG and insulin concentrations (in mg/dL and μIU/mL, respectively) divided by 405 and the QUICKI by the following formula 1/[log(fasting insulin in μIU/mL) + log (FPG in mg/dL)] [13,14].

An oral glucose tolerance test (OGTT) was performed on all participants, with the administration of 1.75 g/kg glucose, with a maximum dose of 75 g. Blood samples were obtained at baseline, 30, 60, 90, and 120 min post-administration to measure insulin and glucose. Matsuda insulin sensitivity index (ISI) was calculated using the following formula: 10,000/square root of [(fasting plasma glucose × fasting plasma insulin) × (mean plasma glucose × mean plasma insulin during OGTT)]. The insulinogenic index (IGI) was calculated as the ratio of (fasting insulin-insulin at 30 min)/(fasting glucose-glucose at 30 min). The disposition index (DI) and oral disposition index (oDI) were calculated using the formulae ISI x IGI and IGI/fasting insulin, respectively [15,16,17].

## 3. Statistical Analysis

The comparisons between the study groups of patients with obesity and the control group and the subgroups were made, respectively, either by the *t*-test and analysis of variance (ANOVA) or with the Mann–Whitney *U* test and Kruskal–Wallis after evaluation of the parameters for normal or not normal distribution. The Benjamini–Hochberg procedure to adjust for the multiple comparisons was also used. A *p* < 0.05 was considered significant, while values were expressed as mean value ± standard deviation (SD) or as median and interquartile range (IR). A sample size of 100 children was found to be appropriate to demonstrate a 20% difference in the variables under examination, with an above 80% power at the level of 0.05 that was set in the present study [18]. The Stat View 5.0 software application of SAS Institute Incorporation (Cary, NC, USA) was used.

## 4. Results

The medical records of 118 children and adolescents potentially eligible for inclusion in the study were retrieved from the registry of the Pediatric Obesity Outpatient Clinic of the University Hospital of Ioannina over a 1-year period. In accordance with the exclusion criteria, 4 youths were excluded from the study, while an additional 10 were not included due to parental refusal to participate.

Of the 104 youths included in the study, 47 (45.2%) were identified as having MHO, while 57 (54.8%) were diagnosed with MUO (Table 1). No statistically significant difference was observed with regard to age, sex, gestational age, birthweight and deviant birthweight, BMI, and pubertal development between the two groups. Regarding the parameters used to discriminate between MHO and MUO, significant differences were found between the two groups of participants. Specifically, the value of TG was significantly lower and HDL-C significantly higher in the MHO compared to the MUO group (91 ± 26 vs. 108 ± 40 mg/dL and 49.8 ± 8.1 vs. 40.4 ± 9.2 mg/dL, respectively). Blood pressure measurements, both SBP and DBP, were lower in the MHO group. There was no significant difference in FG levels between the two groups. There was a trend for waist circumference (WC) to be lower in the MHO group, but this difference was not statistically significant.

The fasting insulin and insulin sensitivity indices, HOMA-IR and QUICKI, were not significantly different between youths identified as having MHO and MUO (2.54 vs. 3.01 and 0.32 ± 0.05 vs. 0.33 ± 0.02, respectively) (Table 2).

The OGTT results were used to assess indices of glucose metabolism in both groups (Table 2). The Matsuda index (ISI) was significantly higher in the MHO group, 4.02, compared to 2.7 in the MUO group (*p* < 0.01), demonstrating greater whole-body insulin sensitivity in individuals with MHO. Furthermore, insulin resistance was demonstrated to be lower in the MHO population by significantly lower values of the insulinogenic index (1.26 vs. 1.54, *p* < 0.01). However, DI and oDI, although not significantly different between the two groups of obese youths, were lower in the MHO group. Since these indices correlate with the body’s response to glucose, with higher values indicating more efficient pancreatic beta cell function, these findings suggest that beta cell function and glycemic control are also affected in children with MHO.

Furthermore, the study population was divided according to pubertal status, and glucose metabolism indices were compared in prepubertal and pubertal individuals classified as having MHO and MUO (Table 3). As expected, insulin 120 was higher in adolescents compared to younger children. In adolescents, insulin 120 and IGI were significantly lower in the MHO group compared to the MUO group (63 vs. 86 μIU/mL and 1.33 vs. 1.7, respectively). However, no significant difference in the above indices or the HOMA-IR and QUICKI indices was observed according to pubertal status. The Matsuda index was significantly higher in the MHO group than in the MUO group in both prepubertal and pubertal subjects. Younger children classified as having MHO showed higher insulin sensitivity compared to adolescents with MHO, as indicated by a significantly higher Matsuda index (4.5 vs. 3.9, *p* < 0.01).

## 5. Discussion

The aim of this study was to compare the glucose metabolism and insulin sensitivity markers between children and adolescents classified as having MHO and MUO. Our findings demonstrate the already described presence of distinct metabolic profiles between the study groups. However, both groups exhibited shared metabolic abnormalities.

A universal definition of MHO in the pediatric population has yet to be established. The first scoping review to propose a universal definition of MHO in children and adolescents was conducted in 2018 by Damanhoury et al. A consensus-based definition of MHO was developed through a Delphi process; however, consensus was not reached on all parameters, namely the measurement of glucose and the inclusion of insulin as a criterion of glucose metabolism [7]. More recently, Abiri et al. also conducted a Delphi process using a panel of experts to develop a consensus-based definition of MHO in the pediatric population [3]. Both consensus groups suggested that weight status should be assessed by WHO BMI-for-age curves and proposed the following criteria to define MHO: high-density lipoprotein cholesterol (HDL-C) > 40 mg/dL, triglycerides (TGs) < 150 mg/dL, systolic BP (SBP) and diastolic BP (DBP) < 90th percentile [3,7]. In addition, Ariti et al. included fasting glucose (FG) < 100 mg/dL in the criteria and the measurement of insulin; however, a consensus was not reached regarding the assessment method for the latter [3].

In this cross-sectional study of a cohort of 104 obese children and adolescents, 45.2% were classified as having MHO and 54.8% as having MUO. Similarly, a recent study conducted in another Mediterranean country using the same diagnostic criteria reported a 49% prevalence of MHO in a cohort of youths with obesity [19]. However, the reported prevalence of MHO varies widely between studies due to the heterogeneity of the classification criteria used and differences in the characteristics of the study populations in terms of stage of pubertal development and ethnicity, with estimates of the prevalence of MHO in pediatric populations with obesity ranging from 3% to as high as 85% [3].

No significant differences were observed between the two groups in regard to sex, BMI, and pubertal status. However, adolescents were less frequently classified as having MHO than as MUO. Regarding WC, in the current study, a trend towards increased WC was observed in children with MUO vs. those with MHO. Higher WC as a surrogate marker of visceral adiposity, systemic inflammation, and metabolic dysregulation has been associated with increased risk of insulin resistance and metabolic syndrome components in children [20]. In addition, it has been linked to a greater risk of future cardiometabolic complications in adult life [21], emphasizing the need for early intervention in such children.

Children in this study with MHO had a more favorable profile, especially regarding the Matsuda index, which was significantly higher in the MHO group, demonstrating greater whole-body insulin sensitivity in individuals with MHO. The Matsuda index is a reliable surrogate marker of the whole body insulin sensitivity, providing a more dynamic picture of glucose-insulin interplay during the OGTT. Its drawbacks include that it assumes a normal β-cell function, which is not always true for children with obesity, and that it has not been standardized across different populations and ages [22] A similar observation was reported by Vukovic et al. in a study comparing children and adolescents with MHO and MUO. Specifically, the Matsuda index was significantly higher in the MHO group (4.43 ± 2.75) compared to the MUO group (2.48 ± 1.61), suggesting preserved insulin sensitivity despite obesity in individuals with MHO [23]. The same authors classified obese children as either insulin-resistant or insulin-sensitive, using either HOMA or Matsuda index values. They observed that, in both classifications, children in the insulin-sensitive group exhibited significantly healthier metabolic profiles [24]. In a more recent study, OGTT was performed in a cohort of youths classified as having MHO or MUO based on the HOMA index or the presence of metabolic syndrome components, and it was concluded that ISI was the strongest predictor of MHO in both classifications [25]. In a study comprising adults with obesity, the Matsuda index was used among other indices to classify MHO vs. MUO, showing that both adults with MHO and MUO had an increased risk of cardiovascular disease, irrespective of the classification criterion [26]. A possible explanation of such a difference in Matsuda index between subjects with MHO and MUO is the observation that individuals with MHO have less ectopic fat (namely, visceral and liver fat) and higher amounts of subcutaneous fat, particularly in the buttocks and legs. This fat distribution pattern is linked to better whole-body insulin sensitivity, less inflammation, and a healthier metabolic profile overall [27].

Moreover, the insulinogenic index was lower in the MHO group, implying high insulin resistance in the MUO population. The IGI, as a measure of the insulin response relative to glucose during the first 30 min of an OGTT, is a marker of β-cell function. It offers important data on early-phase insulin secretion, which is usually the first to be affected during the initial stages of diabetes but does not reflect the overall glucose tolerance [28]. Insulin resistance has been recognized as a significant contributor to the cardiometabolic consequences of obesity. On the other hand, indices such as HOMA and QUICKI, which have been extensively used to estimate insulin resistance status and metabolic syndrome, were similar in both groups. HOMA-IR and QUICKI, which are based on fasting glucose and insulin levels, are simple and widely used indices of insulin resistance, but they reflect basal, not dynamic, states and may be less reliable in populations with extreme insulin resistance or decreased β-cell function [29]. There are few studies that have compared these two indices in children with MUO vs. MHO. In corroboration with our results, Nso-Roca et al., in a study of similar design, found no differences in the HOMA index in two groups of children with obesity with a mean age of 11 years [19]. A number of other studies, as well as an earlier study of our center, have shown similar results [8,30]. Conversely, Genovesi et al., in a larger group of obese children with a mean age of 12 years with either MUO or MHO, found around 25% higher HOMA in the MUO group [5]. Increased insulin resistance, as estimated by the HOMA index, in populations of youths with MUO compared to those with MHO has been demonstrated in several studies [23,31,32].

DI and oDI encompass both insulin sensitivity and insulin secretion in response to glucose, providing a combined measure of how effectively the body responds to changes in blood glucose levels and, thus, can be used as a simple surrogate estimate of β-cell function relative to insulin sensitivity [33]. Both indices did not differ between the two groups of children in the study.

The population of the current study was divided into two groups based on pubertal status, and glucose metabolism indices were compared in prepubertal and pubertal individuals classified as having MHO and MUO. Although not statistically significant, HOMA-IR, insulin 120, and IGI were higher in the pubertal group compared to younger children in both subgroups, MHO and MUO. This is in accordance with the expected appearance of the normal transient pubertal insulin resistance, as a consequence of the hormonal and metabolic alterations that occur during this developmental period [34]. The statistically significant difference observed in the Matsuda index, with higher values in MHO compared to MUO youths, was maintained in both age groups, prepubertal and pubertal. Moreover, prepubertal MHO subjects demonstrated a significantly higher Matsuda index than pubertal MHO subjects, providing evidence of enhanced insulin sensitivity in younger individuals with obesity.

It is important to note that an increasing body of evidence indicates that, despite the reduced risk of complications in comparison to MUO, MHO individuals have a less favorable metabolic status and higher risk of complications compared to those with a normal weight [3,30,35]. A control group of normal weight children was not included in this study. However, the metabolic parameters of individuals with MHO demonstrated a mixed picture, either exhibiting better or similar characteristics to those observed in MUO children.

The term MHO is used to describe the absence of cardiometabolic risk factors at a given time point and is not indicative of a lifetime diagnosis. Indeed, while the concepts of MHO and MUO were initially thought to represent fixed states, an increasing body of evidence suggests that these phenotypes are dynamic, with the potential for individuals to transition back and forth between the two over time. It has been observed that this phenomenon occurs in adult populations, but it is more pronounced in children as they progress through the various developmental stages [4,36]. Reihner et al. examined the transition between MHO and MUO over a one-year follow-up period in a large cohort of obese children, with an average age of 11.6 years, and observed that MHO status was maintained in 68% of youths; however, they reported an association between pubertal status and status transition. In particular, they observed that adolescents at the onset of puberty were twice as likely to switch to MUO, and conversely, adolescents at the end of puberty were three times more likely to switch to MHO [37]. Another large longitudinal study examined the metabolic status of MHO children in adulthood and reported that MHO status was maintained in only 13% of them [38]. However, the recognition of MUO is of considerable importance as it provides an opportunity to identify individuals who would benefit the most from targeted management strategy to prevent the occurrence of metabolic complications [4,7,25].

There are some limitations to this study. Firstly, it is a single-center study, and the sample size is relatively small. Secondly, a group of normal weight children was not included, because OGTT could not be performed without a medical reason. Moreover, although counseling regarding diet and exercise has been offered to all study children during their visits in endocrinology clinics, the details of these two parameters were not recorded. It is true that clamp techniques are the gold standard for estimating β-cell function and insulin sensitivity of individuals. Being cumbersome and invasive, though, they are not easy to perform on a large scale, and therefore, various indices of glucose metabolism were used in the current study. Together, these indices offer complementary insights but vary in complexity, accuracy, and clinical applicability. In addition, they have not been standardized for pediatric populations [39,40]. The strengths of this study are that the groups were homogeneous in terms of sex, age, and pubertal status and that the categorization was done by a senior pediatric endocrinologist.

In conclusion, according to the results of this study, youths classified as having MHO based on the more recent criteria have more favorable metabolic profile than youths with MUO. However, a completely normal laboratory profile was not observed in the MHO group, as many crucial parameters of the metabolic profile were similar to those observed in the MUO group. Based on the results of this study, all children with obesity, regardless of being classified as having MUO or MHO, need close monitoring for both short- and long-term complications.

## Figures and Tables

**Table 1:** Comparison of population characteristics and mean values of parameters used for the discrimination of metabolically healthy obesity (MHO) and metabolically unhealthy obesity (MUO).

Parameters	MHO (n = 47)	MUO (n = 57)
Sex, female	26	31
Age, years	11 ± 2.8	11.5 ± 2.7
Gestational age, weeks	38 ± 1.4	38 ± 1.5
Birth weight, gr	3570 ± 330	3520 ± 280
SGA/LGA	1/9	1/7
Prepubertal/pubertal	22/27	21/36
BMI, z-score	4.1 ± 1.7	4.2 ± 1.7
WC, z-score	3.6 ± 1.7	4.1 ± 2.1
Prepubertal/pubertal	22/27	21/36
HDL-C, mg/dL	49.8 ± 8.1	40.4 ± 9.2
TG, mg/dL	91 ± 26	108 ± 40
SBP, z-score	0.96 (0.51, 1.2)	1.5 (0.9, 1.57)
DBP, z-score	0.61 (0.41, 0.74)	0.88 (0.54, 0.97)
FG, mg/dL	86.9 ± 7	87.4 ± 9

BMI: body mass index; SGA: small for gestational age; LGA: large for gestational age; WC: waist circumference; HDL-C: high-density lipoprotein cholesterol; TG: triglycerides; SBP: systolic blood pressure; DBP: diastolic blood pressure; FG: fasting glucose.

**Table 2:** Indices of glucose metabolism in obese youths with metabolically healthy obesity (MHO) or metabolically unhealthy obesity (MUO).

Parameters	MHO (n = 47)	MUO(n = 57)
Glucose 120, mg/dL	110 ± 21	113 ± 18
Insulin 120, μIU/mL	58 (24, 83) *	74 (33, 99)
Insulinogenic index	1.26 (0.66, 1.8) *	1.54 (1.0, 2.4)
Disposition index	3.8 (2.8, 6.2)	3.56 (2.6, 7.1)
Oral Disposition index	0.13 (0.07, 0.18)	0.11 (0.07, 0.21)
Insulin sensitivity index	4.02 (2.4, 5.3) **	2.7 (1.9, 3.4)
HOMA-IR	2.54 (0.9, 2.99)	3.01 (1.22, 3.43)
QUICKI	0.32 ± 0.05	0.33 ± 0.02
Weight/height	0.88 ± 01	0.90 ± 0.06
Torso/height	0.51 ± 0.03	0.52 ± 0.04

HOMA-IR: Homeostasis Model Assessment of Insulin Resistance; QUICKI: Quantitative Insulin Sensitivity Check Index. * and **: *p* < 0.01 and 0.05 denotes the statistical significance between the MHO vs. MUO groups.

**Table 3:** Indices of glucose metabolism in obese youths with metabolically healthy obesity (MHO) or metabolically unhealthy obesity (MUO) categorized by pubertal status: prepubertal vs. pubertal.

	Prepuberty		Puberty	
Parameters	MHO	MUO	MHO	MUO
Patients	21	20	26	37
Age, years	8.5 ± 2	9.1 ± 2	12.9 ± 1.7	12.9 ± 1.9
Glucose 120, mg/dL	111 ± 22	119 ± 14	108 ± 21	110 ± 19
Insulin 120, μIU/mL	44 (21, 54)	59 (31, 78)	63 (25, 67)	86 (24, 99) *
Insulinogenic index	1.01 (0.65, 1.5)	1.45 (0.6, 2.2)	1.33 (0.6, 1.6)	1.7 (1.1, 2.5) *
Disposition index	3.7 (2.8, 6.28)	3.4 (1.5, 5.1)	3.9 (2.6, 5.4)	4.1 (3, 6)
Oral Disposition index	0.09 (0.07, 0.15)	0.11 (0.05, 0.18)	0.11 (0.07, 0.15)	0.11 (0.09, 0.17)
Insulin sensitivity index	4.5 (2.9, 5.6) **^a^	2.4 (1.8–2.8)	3.9 (2.1, 4,2) *	2.8 (2.1, 3.6)
HOMA-IR	2.4 (1.8, 2.8)	2.6 (1.9, 3.4)	2.8 (2.1, 3.6)	3.5 (1.7, 3.8)
QUICKI	0.34 ± 0.3	0.33 ± 0.1	0.32 ± 0.1	0.33 ± 0.1
Weight/height	0.92 ± 01 ^b^	0.94 ± 0.05	0.86 ± 02	0.89 ± 0.05
Torso/height	0.53 ± 0.02	0.54 ± 0.02 ^a^	0.53 ± 0.05	0.51 ± 0.02
WC, z-score	3.56 ± 1.6	4.1 ± 2.0	3.65 ± 1.8	4.1 ± 2.1

HOMA-IR: Homeostasis Model Assessment of Insulin Resistance; QUICKI: Quantitative Insulin Sensitivity Check Index obesity. * and **: *p* < 0.01 and 0.05 denotes the statistical significance within each pubertal group between MHO vs. MUO ^a, b^ *p* < 0.01 and 0.05 denotes the statistical significance between the prepubertal and pubertal subgroups.

## Data Availability

The dataset is available upon request from the authors.
